# Reliability and validity of the modified Toronto Clinical Neuropathy Score in diabetic sensorimotor polyneuropathy

**DOI:** 10.1111/j.1464-5491.2009.02667.x

**Published:** 2009-03

**Authors:** V Bril, S Tomioka, R A Buchanan, B A Perkins, the mTCNS Study Group

**Affiliations:** University Health Network, University of TorontoToronto, ON, Canada; * Dainippon Sumitomo Pharma, Fort Lee, NJ and Dainippon Sumitomo PharmaAnn Arbor, MI, USA

**Keywords:** clinical score, diabetic neuropathy, diagnosis, modified Toronto Clinical Neuropathy Score, Toronto Clinical Neuropathy Score

## Abstract

**Introduction:**

A reliable and valid clinical tool to capture symptoms and signs of diabetic sensorimotor polyneuropathy (DSP) for use in clinical research trials is urgently needed. The validated Toronto Clinical Neuropathy Score (TCNS) was modified to improve sensitivity to early DSP changes. We aimed to assess the reproducibility of this modified tool, the mTCNS and to determine its validity relative to the precursor TCNS.

**Methods:**

Sixty-five patients (six Type 1, 59 Type 2 diabetes) with diabetes duration 13 ± 8 years were accrued from four study sites and examined on 2 days for internal consistency and inter- and intra-rater reliability of the mTCNS. In the absence of a single quantitative gold-standard measure for DSP, results of the mTCNS were compared with the precursor TCNS for the purpose of estimating validity.

**Results:**

Internal consistency of the two domains within the mTCNS was good (Cronbach's alpha 0.78). Very good inter-rater reliability for the mTCNS was demonstrated by an intra-class correlation coefficient for the mTCNS of 0.87 (95% confidence interval, 0.79–0.91), which was similar in magnitude to that of the TCNS (0.83; 95% confidence interval, 0.75–0.89). Intra-rater reliability testing of the mTCNS showed moderate to good correlation for individual symptoms and sensory tests (Cohen's kappa values of 0.54–0.73). The mTCNS shared moderate correlation with the precursor TCNS (Pearson correlation coefficient, 0.58).

**Discussion:**

The mTCNS, a clinical score with higher face validity for tracking mild to moderate DSP, has sufficient reliability and validity relative to its precursor TCNS for use in clinical research.

## Introduction

Although therapeutic agents such as aldose reductase inhibitors have the potential to restrict the progress of diabetic sensorimotor polyneuropathy (DSP), none have proven efficacious [1]. A possible methodological reason is that clinical assessment of DSP is complex and, as a consequence, biological improvement in nerve function may not be detectable owing to the inherent error variability of the clinical research instruments used to measure DSP. Several instruments measuring similar clinical features to assess patients with DSP have been developed for use in research or clinical practice. For example, the Michigan Neuropathy Screening Instrument (MNSI), used in the study of the aldose reductase inhibitor zenarestat, terminated for reasons of toxicity [2], represents the screening component of a two-step test requiring ultimate confirmation of neuropathy by measurement with the Michigan Diabetic Neuropathy Score (MDNS) [3]. Similarly, a thematically analogous clinical score, the modified Neuropathy Disability Score (NDS), has been used in epidemiological studies of DSP to show association of neuropathy with risk factors [4,5]. Although many other similar scales were concurrently developed [6–8], the search for a clinical scoring system sensitive to the early pathophysiological changes of DSP reversible by disease-modifying therapy such as aldose reductase inhibitors continues.

The Toronto Clinical Neuropathy Score (TCNS; Table 1) can be used to measure changes in such early DSP pathophysiology because of its content validity, and the demonstration of criterion validity against the morphological criteria of sural nerve fibre density for DSP [9,10], and of construct validity against nerve conduction velocities and nerve conduction amplitudes [10]. The TCNS has been preferred in some clinical trials owing to its ease of use, acceptability by patients, its ability to classify the severity of DSP and its representation of the clinical changes associated with the progression of DSP [10,11]. Inherent in these attributes, the TCNS has sufficient reliability and reproducibility as an instrument to document and monitor DSP in the clinical setting [9].

**Table 1:** The components of the original Toronto Clinical Neuropathy Score (TCNS)

Symptom scores	Sensory test scores	Reflex scores
Foot pain	Pinprick	Knee reflexes
Numbness	Temperature	Ankle reflexes
Tingling	Light touch	
Weakness	Vibration	
Ataxia	Position sense	
Upper limb symptoms		
Symptom scores graded as	Sensory test scores graded as	Reflexes graded as
0 = absent	0 = normal	0 = normal
1 = present	1 = abnormal	1 = reduced
		2 = absent

Maximum TCNS is 19 points.

Symptoms and signs (sensory tests) are considered present if because of diabetic sensorimotor polyneuropathy (DSP) in the opinion of the investigator. Details of the TCNS have been published previously [9].

In a nerve biopsy study of the aldose reductase inhibitor ranirestat, with a 15-month extension study, the TCNS showed a statistically significant improvement in the drug-treated patients compared with baseline assessment [11,12]. In this study, improvements were observed primarily in the sensory testing component of the TCNS, but not in the reflex score component. These results using the TCNS suggested that ranirestat could reverse aspects of the pathophysiology of DSP and emphasized the importance of a sensitive clinical scoring system as the outcome measure in therapeutic trials. The TCNS was modified (into the mTCNS, shown in Table 2) to better capture a categorical scale of simple sensory tests, which are better representative of the early dysfunction in DSP, and to eliminate reflex testing, which represent the late-stage pathophysiology of DSP, are highly variable between raters [13], age dependent and heavily weighted in the TCNS [9]. As such, sensory and symptom levels were introduced in the mTCNS to improve sensitivity and specificity of the original TCNS.

**Table 2:** The components of the modified Toronto Clinical Neuropathy Score (mTCNS)

Symptom scores	Sensory test scores
Foot pain	Pinprick
Numbness	Temperature
Tingling	Light touch
Weakness	Vibration
Ataxia	Position Sense
Upper limb symptoms	
Symptom scores graded as	Sensory test scores graded as
0 = absent	0 = normal
1 = present but no interference with sense of well-being or activities of daily living	1 = reduced at the toes only
2 = present, interferes with sense of well-being but not with activities of daily living	2 = reduced to a level above the toes, but only up to the ankles
3 = present and interferes with both sense of well-being and activities of daily living (both)	3 = reduced to a level above the ankles and/or absent at the toes

Maximum mTCNS is 33.

Symptoms and signs (sensory tests) are considered present if as a result of diabetic sensorimotor polyneuropathy (DSP) in the opinion of the investigator.

In view of these potential advantages of the mTCNS over the TCNS for clinical research involving drugs that could modify the early pathophysiology of DSP, we aimed to assess the reproducibility of the mTCNS (measured by inter- and intra-rater reliability). Secondly, in the absence of a single quantitative gold-standard measure for DSP, we aimed to simply validate the mTCNS relative to its precursor, the validated TCNS.

## Methods

We performed a multi-centre study at four centres, three in the USA and one in Canada (see below for list of study centres). The local Institutional Review Board at each of the four sites approved the study protocol prior to enrolment of patients.

### Patients

Sixty-five patients were enrolled (10–22 patients per site). Inclusion criteria included age 18–70 years, presence of diabetes mellitus, a sural nerve amplitude response of 1.0 µV or more and the presence of symmetrical distal DSP as defined by the modified San Antonio Criteria, in which two of symptoms, signs, abnormal nerve conduction parameters or abnormal vibration perception thresholds (VPT) were required [14]. Patients were stratified for disease severity on the basis of the TCNS in order to determine the performance of the mTCNS across the full range of disease severity [9]. Patients were excluded if they had known non-diabetic causes of neuropathy (for example, vitamin deficiencies, uraemia, thyroid disease, lumbar or cervical radiculopathy, inflammatory neuropathy or presence of alcoholism).

### Raters

Each patient was examined on the same day by 1–3 raters for inter-rater reliability testing and then tested again within 48 h by one rater for the intra-observer reliability testing. The raters were all trained at a single training session before starting the study in order to standardize the clinical assessments. Ten raters participated in the study in four study sites.

### Study procedures

At the first visit, demographic information was collected and the TCNS and mTCNS were performed. Details of the TCNS have been presented previously [9]. The mTCNS is a brief, easily administered semi-structured clinical interview and examination during which the trained raters systematically administered the 11 items that assess symptoms and signs of DSP (see Table 2). During the pre-study investigator training session, the symptoms of the mTCNS were described and synonyms suggested to clarify the meaning of the symptoms for patients. Standardized case report forms contained all the elements of the TCNS and mTCNS and were used by the study personnel to complete the scales. The mTCNS rates individual symptoms that are caused by DSP in the judgment of the examiner as absent or present and, if present, graded at levels depending on interference with sense of well-being and/or activities of daily living. Symptoms without interference with sense of well-being or activities of daily living are graded as 1, those which interfere with sense of well-being, but not with activities of daily living as 2, or those which interfere with both as 3. Similarly, each sign as a result of DSP in the judgment of the examiner is rated as normal (0), abnormal at toes only (1), between toes and ankle (2) or above ankle (3) as shown in Table 2.

Other procedures done at the first visit included: MNSI, MDNS, the NDS, nerve conduction studies (NCS) and quantitative sensory testing (QST). NCS included motor nerve conduction studies of the dominant peroneal and tibial and non-dominant median nerves with corresponding F wave latencies and sensory nerve conduction studies of bilateral sural, non-dominant median, radial and ulnar nerves. QST included VPT and cooling detection thresholds measured using the method of limits and the Medoc device (Medoc Advanced Medical Systems Ltd, Durham, NC, USA).

On day 2, each patient had the TCNS and mTCNS performed by 2–4 raters, including the rater from day 1. The assessments were performed at least 15 min apart to reduce memory effects. The time interval of less than 48 h between assessments was selected to minimize the potential for change in symptoms. All examinations were masked to the results of all other testing, including the results of previous TCNS and mTCNS tests. The TCNS was selected as the criterion standard for DSP in this study in the absence of a single, universally accepted gold standard for DSP and the prior demonstration of validity of the TCNS against the morphological criterion of sural nerve fibre density [10].

The NCS, QST, TCNS and mTCNS were reviewed by a central Neurological Core Laboratory for quality control according to established control procedures [15].

To determine the acceptability of the mTCNS to patients, a cognitive debriefing substudy was conducted in 12 randomly selected patients (three from each study site) after the completion of their two study visits. The cognitive debriefing constituted a qualitative semi-structured one-to-one interview that included questions pertaining to the importance of each component of the mTCNS in the opinion of the patients.

### Statistical methods

Internal consistency was assessed for the mTCNS and TCNS using Cronbach's alpha [16]. Inter-rater reliability reflects agreement when the instrument is administered by two or more different raters. Inter-rater reliability was assessed by the intra-class correlation coefficient (ICC) and its 95% confidence interval (CI) from a random effects model in which the patients and raters (*n* = 2–4) were considered random effects. Intra-rater reliability reflects the agreement between responses when the instrument is administered by the same rater on more than one occasion. It was determined by the Cohen's kappa statistic [17,18] for the symptom, sensory test and reflex domains of the mTCNS and TCNS. All raters were combined for calculation of the Cohen's kappa. ICC and kappa statistics were interpreted as very good if 0.81–1.00, good if 0.61–0.80, moderate if 0.41–0.60, fair if 0.21–0.40 and poor if < 0.20 [19]. Validity of the mTCNS was simply assessed according to linear correlation (Pearson coefficients) with the precursor TCNS, but linear correlations with individual measures of DSP, including the results of nerve conduction studies and other objective tests, were calculated.

Construct validity was assessed by correlations of the mTCNS with the TCNS, NCS, QST, MDNS and NDS.

## Results

All 65 patients completed the two visits and all study procedures. Details of their clinical characteristics are shown in Table 3. The majority of the patients had Type 2 diabetes, mean diabetes duration of 13.2 years and mean duration of DSP of 6.6 years. The majority were obese with hypertension. Based on the TCNS, neuropathy was absent in 12.3%, mild in 21.5%, moderate in 27.7% and severe in 38.5%, although all patients met the San Antonio Criteria [14] for DSP. As such, those with ‘absent’ neuropathy according to the TCNS had at least mild degrees of DSP based on the San Antonio criteria.

**Table 3:** Clinical characteristics of the 65 patients with diabetes

Clinical characteristic	
Female sex, *n* (%)	25 (38.5%)
Age (years)	58.5 ± 8.0
Diabetes type	
Type 1 diabetes	6 (9.2%)
Type 2 diabetes	59 (90.8%)
Diabetes duration (years)	13.2 ± 8.0
Race	
Caucasian	48 (73.8%)
African-American	11 (16.9%)
Asian	3 (4.6%)
Other*	3 (4.6%)
DSP duration (years)	6.6 ± 4.4
TCNS (out of 19 points)	10.4 ± 3.8
Severity of DSP according to the TCNS†	
No neuropathy	8 (12.3%)
Mild	14 (21.5%)
Moderate	18 (27.7%)
Severe	25 (38.5%)
mTCNS (out of 33 points)	12.8 ± 6.0
Height (cm)	171.1 ± 9.8
Weight (kg)	103.2 ± 30.9
Body mass index (kg/m^2^)	35.3 ± 10.2
Systolic blood pressure (mmHg)	134 ± 18
Diastolic blood pressure (mmHg)	77 ± 11

Data are presented as *n* (%) or means ± sd.

* Other race includes non-Asian, non-Black/African-American and non-Caucasian patients.

† Diabetes severity graded according to the results of the TCNS. Out of a total score of 19, the grades are defined as follows: 0–5 = no neuropathy; 6–8 = mild neuropathy; 9–11 = moderate neuropathy; ≥ 12 = severe neuropathy.

DSP, diabetic sensorimotor polyneuropathy; sd, standard deviation; TCNS, Toronto Clinical Neuropathy Score.

Ten raters, eight of whom were physicians, participated from the four centres, with a minimum of two raters per site. Three of the raters were neurologists or neurophysiologists, one was an endocrinologist and six had other specialties. The 10 raters had a mean of approximately 3.5–4 years’ experience with the TCNS and other similar clinical scoring systems. They had less experience with the mTCNS (mean 2.6 years). Four (40.0%) raters indicated that the TCNS was the scale with which they were most familiar; other scales were most familiar to 1–3 raters each. The raters had a mean of 11.5 years of experience with neurological examinations and approximately 8 years of experience with monofilament sensory tests and vibration perception threshold testing.

### Internal consistency

The components of both the mTCNS and the TCNS showed good to very good internal consistency. The total scores showed good internal consistency (Cronbach's alpha values 0.78 for the mTCNS and 0.76 for the TCNS). Within the symptom score and sensory test domains, internal consistency was very good for the mTCNS (Cronbach's alpha 0.86 for the symptom score and 0.80 for the sensory test score). Rather than very good, the internal consistencies for the equivalent TCNS domains were good (Cronbach's alpha 0.64 for the symptom score and 0.66 for the sensory test score).

#### Inter-rater reliability

Inter-rater reliability was very good for the total scores of the mTCNS and TCNS and comparable in magnitude (Table 4). The corresponding intra-class correlation coefficients and 95% confidence intervals were 0.87 (0.79–0.91) for the mTCNS and 0.83 (0.75–0.89) for the TCNS. These statistics and the intra-class correlation coefficients for the symptom and sensory test domains are summarized in the first section of Table 4. In the analysis of the symptom score and sensory test domains, the sensory test score had moderate agreement for the mTCNS, but only fair agreement for the TCNS. Measures of inter-rater reliability tended to have higher correlation for the mTCNS as compared with the TCNS.

**Table 4:** Intra-class correlation coefficients (ICCs) and Cohen's kappa statistics for the evaluation of inter- and intra-rater reliability of the mTCNS and TCNS

Parameter	mTCNS	TCNS
Inter-rater reliability (ICCs)*		
Total score	0.87	0.83
Total symptom score	0.92	0.85
Total sensory score	0.52	0.40
Total symptom + sensory score†		0.79
Total reflex score†		0.60
Intra-rater reliability (Cohen's kappas)‡		
Symptoms		
Foot pain	0.73	0.75
Numbness	0.57	0.71
Tingling	0.55	0.62
Weakness	0.64	0.74
Ataxia	0.64	0.75
Upper limb symptoms	0.68	0.79
Sensory tests		
Pinprick	0.56	0.66
Temperature	0.59	0.62
Light touch	0.59	0.69
Vibration	0.54	1.00
Position sense	0.63	0.69
Reflexes†		
Right knee		0.85
Left knee		0.73
Right ankle		0.73
Left ankle		0.76

* All results are based on data from 65 patients evaluated by 10 raters at visit 2.

† Evaluated only for the TCNS.

‡ Cohen's kappa can only be used to analyse discrete parameters because it uses the counts of cases where there is agreement between two assessment times. Therefore, analyses were based only on individual items of the mTCNS and TCNS, not on total scores, which may have had few instances of exact agreement because the divisions are too fine. All results are based on data from 65 patients each evaluated by the same rater at visits 1 and 2 (total of eight raters).

mTCNS, modified Toronto Clinical Neuropathy Score; TCNS, Toronto Clinical Neuropathy Score.

#### Intra-rater reliability

Intra-rater reliability was assessed only for individual symptoms and signs and not the total or domain scores because of the nature of Cohen's kappa that is used to analyse discrete parameters. It uses the counts of cases where there is agreement between two assessment times. Ninety-nine per cent of paired values were concordant within a maximum absolute difference of three points for the mTCNS. For precise concordance of the exact integer scores, generally Cohen's kappa values indicated moderate to very good agreement for the symptoms (Cohen's kappa values of 0.55–0.73) and sensory tests (Cohen's kappa values of 0.54–0.63). Measures of intra-rater reliability were non-significantly higher for the TCNS as compared with the mTCNS and are summarized in the second section of Table 4.

The mTCNS demonstrated moderate correlation with the TCNS (Pearson correlation coefficient of 0.58). Comparison of the symptom score and sensory test domains showed very good and good correlation (symptom score Pearson correlation coefficient, 0.82; sensory test Pearson correlation coefficient, 0.71). Other than the moderate correlation seen between the mTCNS and the MNSI (Pearson correlation coefficient, 0.63), in most cases correlation was poor to fair with other clinical scales. For example, the Pearson correlation coefficient was 0.40 with the MDNS and 0.04 with the NDS. Correlation with individual quantitative objective tests was also poor. For example, correlation was 0.13 with VPT and –0.13 with the summed sensory amplitudes from NCS. However, the Pearson correlation coefficients with the sensory score domain of the mTCNS were higher for these parameters—for example, correlation of this domain with summed sensory amplitude was –0.45. As such, lower (impaired) sensory amplitudes correlated with higher sensory test scores.

The results of the cognitive debriefing indicated that most patients understood the meaning of the questions relating to DSP symptoms that are part of the mTCNS. In the opinion of the 12 respondents, they generally reported that the symptoms in the mTCNS are ‘important’ to ‘very important’ in terms of their relevance to DSP. Details for each symptom in the mTCNS scoring system are shown in Table 5.

**Table 5:** Cognitive debriefing: modified Toronto Clinical Neuropathy Score (mTCNS) symptom importance to patients with diabetic sensorimotor polyneuropathy (DSP)

mTCNS symptom	Important to extremely important	Less than important	Comments
Foot pain	9 (100%)	0 (0%)	Even those who reported no foot pain still indicated that this symptom was ‘extremely important’ to them and should be asked about by physicians.
Numbness	10 (100%)	0 (0%)	
Tingling	9 (90%)	1 (10%)	Patients commented that tingling should be evaluated relative to numbness.
Weakness	8 (89%)	1 (11%)	
Ataxia	7 (78%)	2 (22%)	This symptom was commonly confused with weakness.
Upper limb symptoms	7 (78%)	2 (22%)	

Results given as: number (100%).

Patients were asked: ‘On the following scale, how important is it to you that your diabetes physician asks you about (SYMPTOM)’ where 0 = not at all important, 2 = important and 4 = extremely important.

Twelve subjects underwent a 2-h cognitive debriefing session, but not all questions were explored in all patients.

## Discussion

This study demonstrates that the modification of a well-validated score, designed to capture the manifestations of the early processes of DSP better than the precursor score, produces a measure with similar internal consistency, inter-rater reliability and intra-rater reliability. The resultant measure, the mTCNS, maintains acceptable correlation with the precursor score, the TCNS.

Although this speaks generally to the validity of the mTCNS simply because the TCNS represents well the morphological and functional change in peripheral nerves seen in those with diabetes [9,10], full validation to a quantitative and objective gold standard measure is challenging owing to the complexity of the phenotype of DSP. For example, the criterion standard in clinical research and clinical care for DSP does not consider abnormality of nerve conduction study parameters to be diagnostic of DSP unless accompanied by symptoms or signs [14,20]. Consequently, even our most objective measures for DSP can be considered insensitive for diagnosis and, by extension, for change in the early pathophysiological processes of neuropathy. As such, we did not anticipate strong correlation between the mTCNS and individual nerve conduction study parameters or with the results of other individual objective tests. However, sufficient correlation with the parameters was observed with the most sensitive objective measures, the electrophysiological parameters representing sensory nerves. In particular, the sensory test domain of the mTCNS shares moderate correlation with the sensory nerve conduction study parameters and is consistent with earlier reports of construct validity for the precursor score, the TCNS [10]. In that previous work, the NCS showed Pearson correlation coefficients in the range of 0.3–0.5 with the TCNS for summed nerve conduction velocities, summed amplitudes and sural nerve fibre density [10]. The difference in correlations with NCS between the scales is likely as a result of the exclusion of the tendon reflex scores from the mTCNS, which generally contribute insensitive components for the assessment of early DSP.

Although the mTCNS correlates with the TCNS and other clinical scales measuring DSP, in many cases the degree of correlation is lower than would be expected from scales that make use of similar symptoms and signs. For example, MNSI shares a Pearson correlation coefficient of 0.63 with the mTCNS, even although they have in common similar elements on the semi-structured questionnaire and examination. However, perfect correlation between the mTCNS and the other clinical scales (the TCNS, NDS and MNSI) would indicate that the new modified scale would offer no potential advantage over the existing metrics for neuropathy. As such, the degree of correlations observed might indicate advantages of the mTCNS over the other scales that generally include variables associated with advanced neuropathy and may therefore be less sensitive to milder degrees of change in nerve function. Specifically, the MNSI includes examination of the feet for deformities and reflexes (not components of the mTCNS), while the NDS excludes assessment of symptoms in place of reflexes.

Similarly, correlation with specific quantitative sensory tests (cold detection thresholds) may also relate to the inability of this test to represent the full DSP phenotype as well as a scale that includes more sensitive components such as a quantitative representation of symptoms. Furthermore, cold detection thresholds are characterized by significant error variability that may also explain poor correlation with the mTCNS.

The reproducibility of a quantitative measure for DSP is of fundamental importance as a tool for identifying changes in DSP severity that are of small magnitude. The very good inter-rater reliability of the mTCNS in this study (Kraemer's kappa 0.87) is comparable with that reported previously from a study of the TCNS [9]. That the inter-rater reliability is consistent between these two studies is fundamentally important because the current study is a multi-centre study involving four different centres and 2–4 different examiners at each site, while the latter study that established the TCNS as a reproducible measure was conducted at a single centre [10].

Although serving to quantify the reliability of the mTCNS and to establish its validity in a patient group with mild to moderate DSP severity, this analysis has some limitations. First, the assessment of any proxy score for DSP is hindered by the lack of a single quantitative gold standard measurement for neuropathy. As such, the validity of the mTCNS can only be inferred by its association with the TCNS and components of objective tests. Second, although performance of the mTCNS was generalizable among study centres by different study staff, these centres represent a certain expertise that may limit our knowledge of the applicability of this test in broader clinical settings, such as in primary care clinics.

Taken together, the results of this multi-centre study have implications for future treatment trials in DSP. Patients in this study were selected for mild to moderate levels of DSP as evidenced by the presence of bilateral sural nerve responses. This feature of the study group makes it similar to the patient populations in prior therapeutic studies of agents such as aldose reductase inhibitors [11,21]. As clinical studies of such disease-modifying drugs are expected to be, by necessity, long-term trials [1], highly reproducible summary scores for DSP are required. The mTCNS appears to have the necessary reliability measures for inclusion as an important clinical outcome in these trials.

## Competing interests

VB and RAB have been reimbursed by Dainippon Sumitomo Pharma for consultation fees. ST is an employee of Dainippon Sumitomo Pharma USA. BAP has nothing to declare.

## mTCNS Study Group and study centres

The mTCNS Study Group consists of V. Bril (University of Toronto, Toronto, Canada), A. Shaibani, S. Schwartz and R. Weinstein. The four study sites were University Health Network, Toronto, Canada, Nerve and Muscle Center of Texas, Houston, Diabetes and Glandular Disease Research Associates, San Antonio, Texas and Diablo Clinical Research, Walnut Creek, California, USA.

## Abbreviations

DSP: diabetic sensorimotor polyneuropathy
ICC: intra-class correlation coefficient
MDNS: Michigan Diabetic Neuropathy Score
MNSI: Michigan Neuropathy Screening Instrument
mTCNS: modified Toronto Clinical Neuropathy Score
NCS: nerve conduction studies
NDS: Neuropathy Disability Score
QST: quantitative sensory testing
TCNS: Toronto Clinical Neuropathy Score
VPT: vibration perception thresholds
